# Mouse Resistin (mRetn): cloning, expression and purification in Escherichia coli and the potential regulative effects on murine bone marrow hematopoiesis

**DOI:** 10.1186/s12896-015-0221-1

**Published:** 2015-11-16

**Authors:** Fangyuan Wang, Jin Gao, Alyssa Malisani, Xiaowei Xi, Wei Han, Xiaoping Wan

**Affiliations:** The Center of Research Laboratory, The International Peace Maternity and Child Health Hospital, School of Medicine, Shanghai Jiaotong University, Shanghai, 200030 China; Department of Obstetrics and Gynecology, Shanghai Jiao Tong University Affiliated First People’s Hospital, Shanghai, 200080 China; School of Pharmacy, Shanghai Jiao Tong University, Shanghai, 200240 China; College of Pharmacy, Washington State University, Spokane, WA 99202 USA; College of Arts and Sciences, Gonzaga University, Spokane, 99258 USA; Department of Obstetrics and Gynecology, Shanghai First Maternity and Infant Hospital, Tong Ji University School of Medicine, No.536, Changle Road, Shanghai, 200080 China

**Keywords:** Retn, Recombinant protein, Expression, Inclusion bodies, Purification, Bone marrow regeneration

## Abstract

**Background:**

Resistin (Retn) is a cytokine which has a controversial physiological role regarding its involvement with obesity and type II diabetes mellitus. Recently, murine Retn was found to be a possibly potential regulator of hematopoiesis in mice shown in the screening results of a set of gene chips which mapped the expression level of murine genes during regeneration of impaired bone marrow (BM) by 5-fluorouracil.

**Results:**

Recombinant mice Retn was expressed in Escherichia coli and purified using ion exchange chromatography. Totally 11.4 mg rmRetn was obtained from 500 ml culture with endotoxin level less than 1.0 EU/ug. The purity of recombinant murine Resistin reached to at least 97.6 % via SDS-PAGE analysis and HPLC. The protein possessed chemotaxis effects in the mouse aortic endothelial cells in vitro in transwell analysis. In vitro, rmRetn could up regulate the CFU number of mice BM and after rmRetn was administered, the cell number of murine bone marrow was significantly increased in vivo after chemotherapy. Finally, rmRetn was found able to protect mice from the chemotoxicity of 5-fluorouracil.

**Conclusions:**

The discovery demonstrated a new function of murine Retn and suggested that it could potentially accelerate bone marrow regeneration post chemotherapy.

## Background

Identified in 2001 [1] as a new adipocytokine, Resistin (Retn) is either known to be found in inflammatory zone 3 (Fiz3) as an adipocyte-specific secretory factor (ADSF). The protein was named Retn because in many murine obesity models, the circular level of Retn increases, inducing insulin resistance [2–6]. Retn, a cysteine-rich protein secreted by the adipose tissue of rodents or by the immune and epithelial cells of mammals, has a high sequence identity among species [7, 8]. In rodents, members of the same family are mouse Retn-like alpha (Retnla), beta (Retnlb) and gamma (Retnlg) [1, 9, 10]. However, only two of these analogs of the family, RETN and RETNLB [11] were recognized in human beings, which showed significant sequence homogeneity from its murine orthologs [12]. Recently Retn was found in energy metabolism and inflammatory response, which suggests that Retn may act as a bridge linking the inflammation and insulin resistance [13]. In vitro studies suggest that Retn acts as a pro-inflammatory cytokine which activates endothelial cell functions inducing proliferation and migration of smooth muscle, and promoting macrophage lipid deposition. This results in lipid metabolism and other processes which facilitate the occurrence of atherosclerosis and its development.

Besides the functions mentioned above, our lab accidentally found Retn to exert effects on bone marrow regeneration from the toxicity of chemopeutical agents. Therefore, a hypothesis was suggested that there is a strong relationship between Retn expression and damage or regeneration of bone marrow. Consequently, this meant Retn may play a critical role in bone marrow regeneration after damage, and eventually protect the impaired tissues. It was obvious that it would be meaningful to verify the new function of Retn and that it might lead directly to a potential candidate or target for relieving the bone marrow suppression by chemotherapeutic agents, which is one of the main clinical side effects of chemotherapy.

Given the potential value of the hypothesis, it is necessary to determine whether mouse Retn can protect bone marrow from the damage of chemotherapeutic agents in mouse models. For this reason, an abundance of high quality and suitable murine models with impaired bone marrow was prepared in advance.

In the present work, a feasible and cost-effective preparing method for rmRetn was developed and an appropriate murine model of impaired bone marrow was introduced to validate the newly discovered effect of mRetn on hematopoiesis.

## Methods

### Ethics statement

Our animal research carried out was in accordance with the recommendations in the Guideline for the Care and Use of Laboratory Animals of China. The protocol was approved by the Committee on the Ethics of Animal Experiments of school of pharmacy in Shanghai Jiaotong University. All efforts were made to minimize animal suffering.

### Expression profile of mRetn in gene chip

In order to find proteins that exert the most effect on bone marrow regeneration from the toxicity of chemopeutical agents, a group of 430 2.0 Affymetrix mouse genome probe sets containing 39000 transcripts or 14000 genes, were screened. The hybridized total RNA of mouse bone marrow was extracted at different times post chemotherapy by 5-fluoracil in Balb/c mice.

### ELISA of mouse Resistin

Mouse peripheral blood (PB) samples were obtained by retro-orbital puncture. Each blood sample was coagulated in the refrigerator at 4 °C, centrifuged at 1000 xg for 15 min. The serum was collected and stored at −70 °C. Aliquots were used once only for the test and were not subjected to repeated freeze−thaw cycles. Murine Resistin enzyme-linked immunosorbent assay (ELISA) kits were used to measure Resistin serum levels according to the instructions of the manufacturer.

### Construction of the rmRetn expression plasmid

The mRetn coding sequence was cloned from the cDNA, reversely transcribed from total mRNA of Balb/c mouse liver tissues. The fragment was then amplified by PCR using sense primer 5’-CATGCCATGGGGTCCAGCATGCCACTGT-3’ and antisense primer 5’-CCCAAGCTTTCAGGAAGCGACCTGCA-3’. The Xho I and EcoR I sites (underlined) were incorporated into primers to facilitate directional cloning of the PCR product into the polyclonal sites of the expression vector pET28a(+) (Novagen, USA). PCR was performed in the following conditions: 94 °C for 2 min; 94 °C for 30 s, 55 °C for 20 s, 72 °C for 20 s, 32 cycles; 72 °C for 10 min. The column-purified PCR product and vector was digested with Xho I and EcoR I (Fermentas, Canada), purified with agarose-gel, and ligated together. E. coli strain DH5a (Novagen, USA) was chemically transformed with the recombinant vector and cultured at 37 °C on Luria-Bertani (LB) agar plates with kanamycin (0.1 mg/ml) in order for a selection. Plasmid pET28a-rmRetn was constructed and analyzed by restrictive enzyme digestion and finally confirmed by DNA sequencing (Genscript, China).

### Expression of rmRetn

The recombinant strain E. coli BL21 (DE3) (Novagen, USA) harboring the plasmid pET28a-rmRetn was cultured in 37 °C LB liquid medium containing 0.1 mg/ml kanamycin, shaking at 250 rpm for 12 h. The culture was sub-cultured into fresh LB medium at a ratio of 1:10 to the final medium volume under the same condition. When the optical density (OD) of culture reached 0.8 at 600 nm, isopropyl-β-D-thiogalactopyranoside (IPTG) (Merck, Germany) was added into the medium making a final concentration of 1 mM in order to induce rmRetn expression. The incubation was continued for another 8 h at 42 °C, and cells were harvested by centrifugation at 12 000 rpm for 15 min and washed with phosphate-buffered saline (1× PBS, 137 mM NaCl, 2.7 mM KCl, 4.3 mM Na_2_HPO4, 1.4 mM KH_2_PO4; pH 7.4).

### Isolation, denaturation and refolding of rmRetn inclusion body

10 ml ultrasonication buffer (1 × PBS, 1 mM EDTA, 0.1 mM PMSF; pH 7.4) was added to resuspend 0.5 g cell paste. The suspension solution was then sonicated, (Scientz, Ningbo, China) with 5 s working and 5 s resting in ice water as one cycle for 10 min in total. Then bacterial inclusion bodies were collected by centrifugation at 12 000 rpm for 15 min at 4 °C. Then they were washed once with PBS and centrifuged, and inclusion bodies were resuspended in denaturing buffer (6 M guanidine chloride, 1 mM EDTA, 50 mM NaCl, 50 mM Tris–HCl) at a ratio 10:1 (mg:ml) and shaken for about 2 h until the inclusion body was dissolved thoroughly. After centrifugation, the protein in the supernatant was refolded by a slow dilution of the denaturing buffer into the renaturing buffer (1 mM reduced glutathione, 0.1 mM oxidized glutathione, 0.5 M guanidine chloride, 0.4 M sucrose, 0.1 M Tris–HCl; pH 6.0) of 100 volumes of the denaturing buffer with gentle shaking at room temperature for 1 h and then followed by another 1 h shaking after dilution. After centrifugation at 15 000 rpm for 30 min, the supernatant containing the renatured protein was prepared for the following purification.

### Purification of rmRetn

The protein in the dilution buffer was loaded continuously onto a 20 ml Q sepharose column and then a 20 ml SP Sepharose column, which were well prequilibrated with 20 ml buffer A (20 mM Tris–HCl, 1 mM EDTA, 25 mM NaCl; pH 6.0), at a speed of 1 ml/min. After the cation exchange column was removed away, the protein was washed with 40 ml buffer A, and then eluted using a 0–1.0 M NaCl gradient in the same buffer with a speed of 1 ml/min. Fractions were collected according to their UV absorption peaks and protein concentrations were quantified using the Bradford method [14].

### SDS-PAGE assay and western blotting

SDS-PAGE was performed using a 15 % running gel on the Power-Pac Basic (Bio-Rad, USA). Briefly speaking, the protein samples were loaded on the gel and were electrophoresed at 120 V for 1.5 h. The bands of proteins on the gel were visualized by staining with Coomassie brilliant blue R-250. In Western blotting experiments, proteins were transferred onto polyvinylidene fluoride (PVDF) membranes at 200 mA for 60 min. The membranes were first blocked in PBS containing 10 % (w/v) non-fat milk, and then incubated with a polyclonal antibody of mouse Retn (R&D, USA), diluted at 1:2000 in PBS containing 0.05 % Tween 20 (PBST) and 5 % (w/v) non-fat milk. After a washing, the membrane was incubated in the presence of horseradish peroxidase (HRP) conjugated secondary antibody (Jackson immuno, USA) diluted at 1:2 000 in PBST with 5 % (w/v) non-fat milk powder. All the incubating procedures were performed at 37 °C for 1 h or 4 °C for overnight with gentle shaking and the membranes were washed for three times before each incubation. Finally, the membrane was thoroughly rinsed five times with PBST for 5 min each, and then the rmResitin protein was visualized through enhanced electrogenerated chemiluminescence (ECL) reaction on X-ray film using western blotting substrate reagent (Thermo, USA).

### HPLC analysis

RP-HPLC was performed to determine the purity of rmResistin with an ODS C18 column (4.6 × 250 mm, 5 μm) on Shimadzu LC 2010A HT (Shimadzu, Japan). Twenty five microliters of rmRetn at 1 mg/ml in PBS was loaded. The protein was eluted with acetonitrile solution at a speed of 1 ml/min using an isocratic program at a constant 70 % acetonitrile. The purity of the protein was calibrated by the area normalization method of OD 280 peaks. SEC-HPLC was performed to determine the purity and polymers of rmResistin using TSK-GEL G2000SWXL (Tosoh, Japan) on ACME 9000 (Younglin, Korea). 50 μl of rmRetn at 0.5 mg/ml in PBS was loaded. The protein was eluted with phosphate buffer (1.27 mM Na_2_HPO4, 1.5 mM KH_2_PO4, 0.4 M NaCl; unadjusted pH) at a speed of 0.8 ml/min. Absorbance at 280 nm was used to monitor the elution profile.

### Endotoxin assay

The endotoxin level of rmRestistin solution was determined semi-quantitatively using the Chromogenic Tachypleus Amebocyte Lysate (TAL) end-point assay kit according to the protocol given by reagent supplier (Xiamen Houshiji, Ltd., Xiamen, China). The assay was the pharmaceutical industry standard procedure for quality control of biologics.

### Bioactivity assay

Bioactivity of rmRetn was determined using a modified Boyden chamber assay [15], following the protocol provided by BD company. Balb/c mouse aortic endothelial cells (MAECs) were isolated and cultured as reported previously [16]. Prior to each experiment, cells were subjected to serum starvation in routine medium with only 1 % fetal bovine serum (Gibco, USA). The experiments were performed as following: cell suspension (250 μl, 106 cells/well) was transferred to the transwells (Pore size 8 um, Costar, MA), and then, 750 ul starvation medium (supplanted with 1 % fetus bovine serum) with or without rmRetn. The wells were incubated at 37 °C with 5 % CO2 atmosphere for 5 h. The migratory cells were qualified by counting five random microscopic fields (×40) after dying with crystal violet. The assays were performed in triples and the results were expressed as a ratio of the total number of migrated cells in all five fields of each well compared to the control.

### BM CFU assay in vitro

In vitro bioactivity of the protein was detected by measuring its BM cell proliferation-stimulating activity. Briefly speaking, BM cells were obtained from the BM of male SPF BALB/c mice as previously described [17], and then added to culture dishes with methyl cellulose medium (containing fetal calf serum, glutamine, β-mercaptoethanol and IMDM), mixed with cytokines (mSCF, 10 ng/μL; mIL-6, 10 ng/μL; mIL-3, 10 ng/μL; mTPO, 10 ng/μL; hG-CSF, 20 ng/μL; hEPO, 10 000U/mL, R&D systems, USA) and different concentrations of rmRetn. PBS was used instead of rmRetn in the control group. After incubated for 7 days at 37 °C with 5 % CO2, the dishes were observed under a microscope, and the colonies (clusters with more than 50 cells are defined as colonies) were counted. The assay was performed in triples and the results were expressed as total number of CFU on each plate.

### Mouse hematocyte instimulating assay

In vivo bioactivity of rmRetn was determined as follows: Male BLAB/c mice aging 6–8 weeks (*n* = 64) were purchased from Shanghai Slac Laboratory Animal Co. (Shanghai, China). Mice were maintained under standard conditions and received humane care in accordance with protocols approved by Shanghai Jiao Tong University and the legal requirements in China. The mice were allowed to adapt to their environment for 1 week before experiments. The mice were randomly assigned to 4 groups, with 16 animals in each group. In the experiment group, the rmRetn was diluted in sterile PBS to a concentration of 1, 10 and 100 μg/mL respectively, before it was used. The mice in three experiment groups received single intraperitoneal injections of 100 ul at doses of 5, 50 and 500 μg/kg body weight of rmRetn, respectively. The mice in the control group were intraperitoneally injected with the same volume of PBS. The mice received the injection of rmRetn or PBS every 12 h for 168 h. Two time points during or post rmRetn administration (that is day 5 and 10) are selected for counting BM cells of mice.

### Mouse survival rate protection assay

Male BLAB/c mice aging 6–8 weeks (*n* = 45) were used in this experiment extending for two weeks. Mice were randomly assigned to 3 groups with 15 animals in each group. Mice in the first group were treated with single intraperitoneal injection of rmRetn (250 μg/kg weight) from day 0 to 4, once per day, and 5-Fluorouracil (300 mg/kg weight) were injected at day 5. Mice in the second group were injected with 5-Fu (300 mg/kg weight) only. For the third group, mice were injected intraperitoneal of 5-Fu (300 mg/kg weight) first at day 0 and rmRetn (250 μg/kg weight) was used from the second day to sixth day. The mice survival conditions were recorded and analyzed to investigate the effect of rmRetn on the survival rate of mice.

### Statistical analysis

All data was expressed as the mean ± Standard Deviation (SD). The P values at the same time point are calculated by one-way analysis of variance followed by 2-tailed Student’s *t*-test. The difference of P values with less than 0.05 was considered significant in all experiments and is indicated by symbols and the difference of P values with less than 0.01 was considered extremely significant. Survival analysis was performed using log-rank test and expressed as Kaplan-Meier survival curves. All statistical analysis was performed using Statistical Product and Service Solutions (SPSS; version 18.0) software (IBM, USA).

## Results

### 5-Fu treatment induces expression of Retn in BM cells

**Fig. 1:**
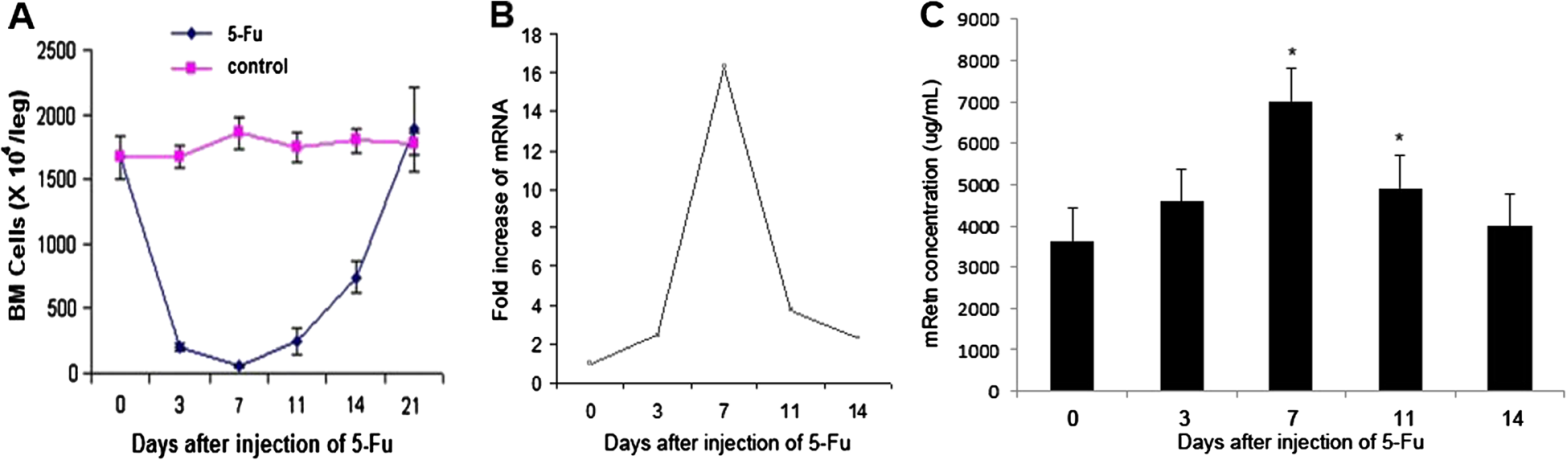
Upregulation of Retn in BM cells after ablation. **a** Normal Balb/c mice were injected with 5-Fu (300 mg/kg) or PBS as control at day 0. Total BM cell numbers per leg decreased remarkably post 5-Fu injection compared to the control (*P* < 0.001). 6 mice (3 males and 3 females) were individually investigated at each time point in 3 independent experiments. **b** Gene expression analysis in the Affymetrix oligonucleotide microarrays. Total mRNA of BM cells of mice was extracted at day 0, 3, 7, 11 and 14 post 5-fluorouracil injection and mixed thoroughly for genechip hybridization. 6 mice were utilized at every time point after chemotherapy, respectively. Microarray hybridizations were performed on gene chip Affymetrix mouse genome 430 2.0. The hybridization intensities of Retn were shown at 0, 3, 7, 11, 14 days after 5-Fu treatment. The mRNA level of mResistin reached the maximum of 16.3 fold of the normal level on the 7th day after chemotherapy. **c** Serum samples obtained from PB were assayed for Retn by ELISA. Samples of 3 mice at each time point were analyzed individually, and great increase was detected by day 7 (*P = 0.008) and 11 (*P = 0.003) after chemotherapy, compared with the basic concentration at day 0. All values were given as mean ± SD

In order to identify candidate genetic molecules contributing to the BM damage and regeneration, BM suppression was induced by a single injection of 5-Fu to normal mice. Total BM cells reached the minimum at day 7, then returned to normal level at day 21 post 5-Fu treatment (Fig. 1a).

mRNA expression in total BM cells was profiled using microarray. Immediately after impair, 567 of the 14000 detected genes were found to be increasingly expressed and then they decreased, whose products was considered as the most likely target proteins which were highly expressed to initiate and promote the recovering progress of bone marrow. As a member of the gene clusters, mRNA of Retn increased more than 2 folds compared to the control in 3 days post 5-Fu injection, and then it continued to increase more than 16 fold compared to the control until 7 days later when it started to gradually decrease to its normal level (Fig. 1b). Such a great change only happened in rare genes.

Increases in the level of plasma Retn were observed at day 7 (*P* = 0.008) and 11 (*p* = 0,026), recovering to pretreatment levels at day 14 following 5-Fu (Fig. 1c). The expression profiles of Retn correlated inversely to the BM cellularity after 5-Fu treatment, which suggests that Retn may play an important regulatory role in BM homeostasis.

### Expression of the mature form of rmRetn in E. coli

**Fig. 2:**
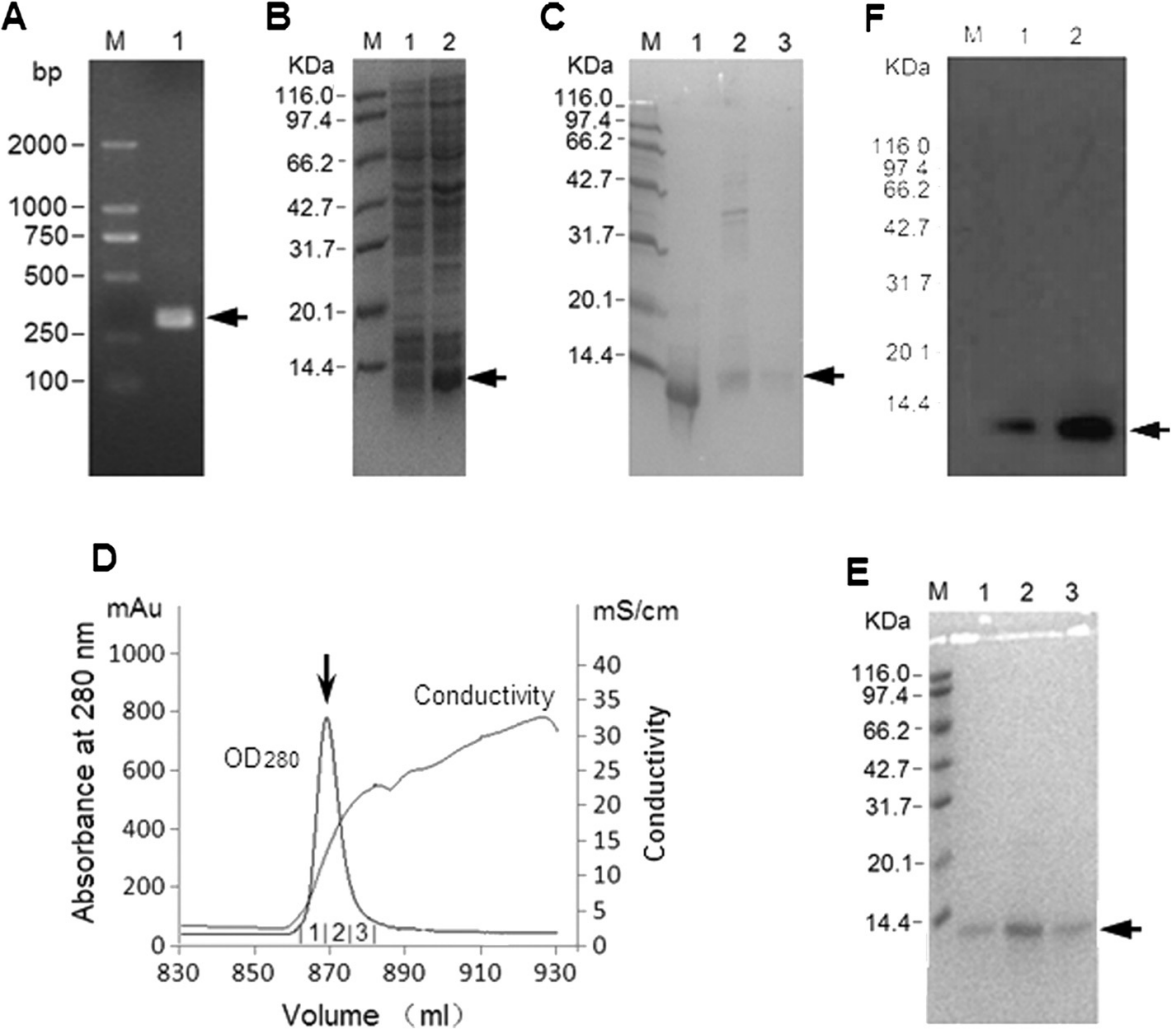
Cloning, expression, purification and characterization of rmResistin. **a**: Construction of rmRetn expression plasmid, and *arrow* indicates the PCR product, of mResistin ORF, which contains 288 base pairs and codes 94 amino acids of mature mResistin with an additional initiating codon on 5’ terminus and a stop codon on 3’ terminus. M, marker; 1, PCR product of rmResistin expression plasmid verification. **b**: Compared with whole bacteria proteins without induction, a protein band, as *arrow* indicates, appears after induction with IPTG. M, marker; 1, whole bacterial proteins prior to induction; 2, whole bacterial proteins 4 h post induction with 1 mM IPTG. **c**: Inclusion bodies of rmResistin were dissolved in denaturing buffer of GdnHCl (lane 1) and renatured in renaturing buffer (lane 3). The impurities and unsuccessfully renatured inclusion bodies (lane 2) were removed. M, marker; 1, solubilized inclusion bodies; 2, pellet of centrifugation post renaturation; 3, the renatured protein solution. **d**: Loaded on the SP sepharose Fast Flow resins, rmResistin was washed and eluted with linear NaCl concentration from 0.02-1 M and the only UV280 peak, eluted out when conductivity reached, as the *arrow* indicates, shows rmResistin. Absorbance at 280 nm and conductivity were detected on monitor. The elution was fractioned into fraction 1, 2 and 3. **e**: Three fractions were subjected to 15 % SDS-PAGE and only one band with molecular weight about 10.4 KDa was observed in the three lanes; M, molecular weight marker; 1, fraction 1; 2, fraction 2; 3, fraction 3. **f**: Western blotting results of rmResistin. Only one band positioned at about 10.4 KDa was obverved and this was identified as rmRsisitin monomer. No dimmers, trimers or polymers of rmResistin were found. M, Molecular weight standard marker; 1, whole bacterial proteins containing induced rmresistin by IPTG; 2, purified rmResistin

The full length of mouse Retn cDNA is 1139 base pairs (bps) with a coding sequence (CDS) of 345 bps positioning from 85 to 429 bp, encoding the full length protein (GeneBank accession no. NM_022984.4). The first 60 bps of CDS encode a signal peptide containing 20 amino acids, which needs to be removed for maturation, so the mature form of mouse Retn contains 94 amino acids. Accordingly, the recombinant mRetn (rmRetn) expressed in prokaryotic expression system will consist of 95 amino acids, coded by 285 base pairs, with an additional Met at N-terminus. The theoretical pI of both mRetn and rmRetn is 8.06. The amplified fragment from the cDNA library was verified on agarose gel (Fig. 2a). Subcloned into E. coli expression vector pET28a, the fragment sequence was confirmed again by DNA sequencing.

After transformation, E. coli strain BL21 (DE3) harboring plasmid pET28a-rmRetn,cultured in LB liquid medium and induced by IPTG, produced a protein positioned at about 10 KDa site on SDS-PAGE. The molecular weight was 10.3 kDa which was calibrated based on the shift rate of the marker bands, exactly equal to the theoretical molecular weight of rmRetn (Fig. 2b). The western blotting further indicated that the protein was rmRetn which was expected. After sonication and centrifugation at high speeds, the protein was found to exist only in a pellet, which suggested that rmRetn was expressed only in the form of inclusion bodies. The following temperature adjustment during induction didn’t change the situation and just affected the amounts of expression. Finally, the induction was performed under 42 °C for 5 h since the expressing amount did not increase over a longer period of time and then rmRetn was purified from inclusion bodies.

To prepare enough inclusion bodies, 500 ml LB medium culture of E. coli BL21 (DE3) harboring pET28a-mRetn were induced under the optimized condition, totaling 200 mg (wet weight) inclusion bodies obtained.

### Denaturing and refolding results

100 mg inclusion bodies were thoroughly solubilized in 10 ml denaturing buffer, and only a little pellet of precipitation was found after centrifugation. The protein in clear supernatant was refolded by gradual dilution into a renaturing buffer at 0.2 ml/min, nearly half (45 %) of the inclusion bodies were refolded successfully and they turned out to be soluble after centrifugation (Fig. 2c).

### Ion-exchange chromatography result

Diluted in the same volume of balance buffer, rmRetn was loaded onto an anion-exchange (Q sepharose) column and then a cation-exchange (SP Sepharose) column. The protein was eluted out directly by a binding buffer and was bound on SP Sepharose resin. A single peak of rmRetn was eluted out by increasing the gradient salt when conductivity reached 30 mS/cm, corresponding to 0.684 M NaCl, after the signal of impurities reached zero (Fig. 2d). The fractions collected under monitoring were analyzed on SDS-PAGE and then combined, and finally 1.8 mg rmRetn was obtained in 4.2 ml elution buffer (Fig. 2e).

### Characterization and quality analysis of purified rmRetn

The final product could be decided undoubtedly to be rmRetn because the cDNA had been sequenced correctly and the actual molecular weight on SDS-PAGE was unanimous with calculations. Furthermore, the protein could also bind to an anti-mRetn antibody in the western blotting assay (Fig. 2f).

**Fig. 3:**
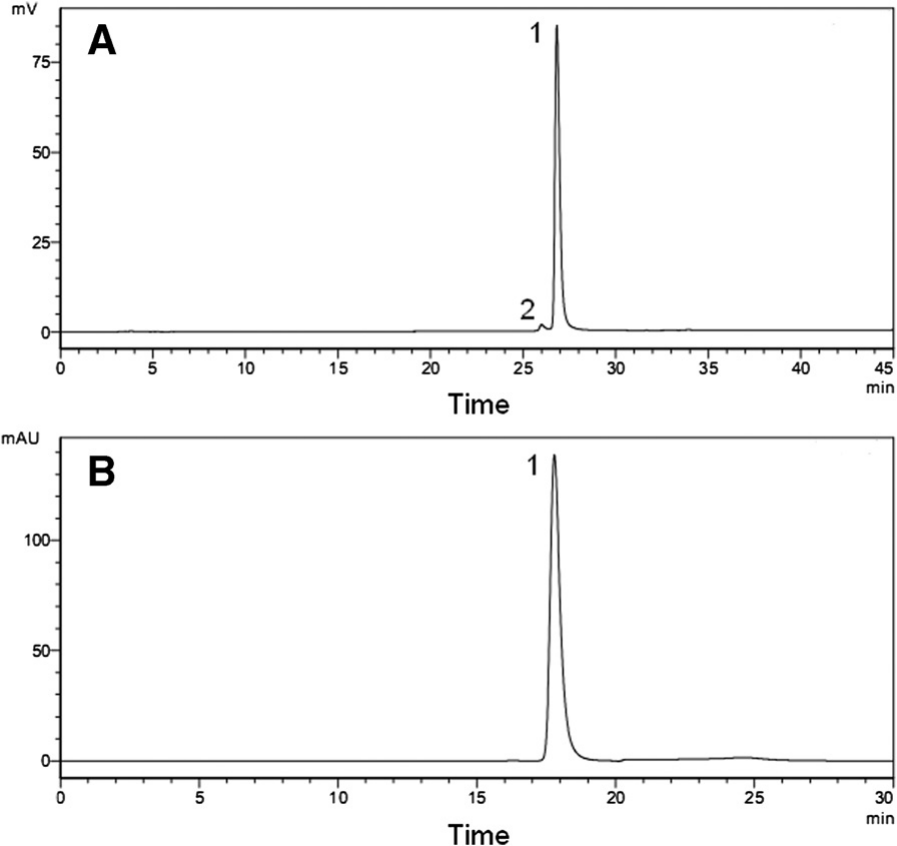
HPLC analysis results of purified rmRetn. **a**: RP-HPLC analysis of purified rmRetn was employed to detect sulphoxides, deamidates and/or impurities. Totally 28.5 μg rmRetn in 30 μl was loaded for analysis and two peaks were observed in the RP-HPLC spectrum, with the large one (1) identified as rmRetn and minor one (2) as other material. The purity by RP-HPLC was 97.6 %; **b**: SEC-HPLC was performed to detect possible polymers of rmRetn. Totally 47.5 μg rmRetn in 50 μl was loaded for analysis and only one rmRetn peak (1) was checked in SEC-HPLC spectrum and the purity of rmRetn was 100 %. Both spectra were detected at ultra violet (UV) wave length 280 nm and had been divided by blank control spectra respectively

The quality was evaluated mainly through purity and endotoxin analysis. On the SDS-PAGE gel, there was only one band that could be seen, which indicated that the recombinant protein was already very pure. The purity was further analyzed by RP-HPLC and SEC-HPLC, and a different material, occuping 2.4 % of total peak area, was detected on RP-HPLC spectrum. Thus, the purity of rmRetn was determined to be 97.6 % (Fig. 3a). The SEC-HPLC analysis identified only one peak of rmRetn and no multimers of it, for example the trimers and hexamers that had been reported in the past articles [18, 19] (Fig. 3b). As a result, 4.7 mg in 13 ml and 6.7 mg in 7 ml of purified rmRetn were recovered after the combination of fraction 1 and 3. Endotoxin of the purified rmRetn, previously determined as prescribed, was less than 1 EU/μg, which was considered to be acceptable for animal use [20].

### Bioactivity analysis result of rmRetn

**Fig. 4:**
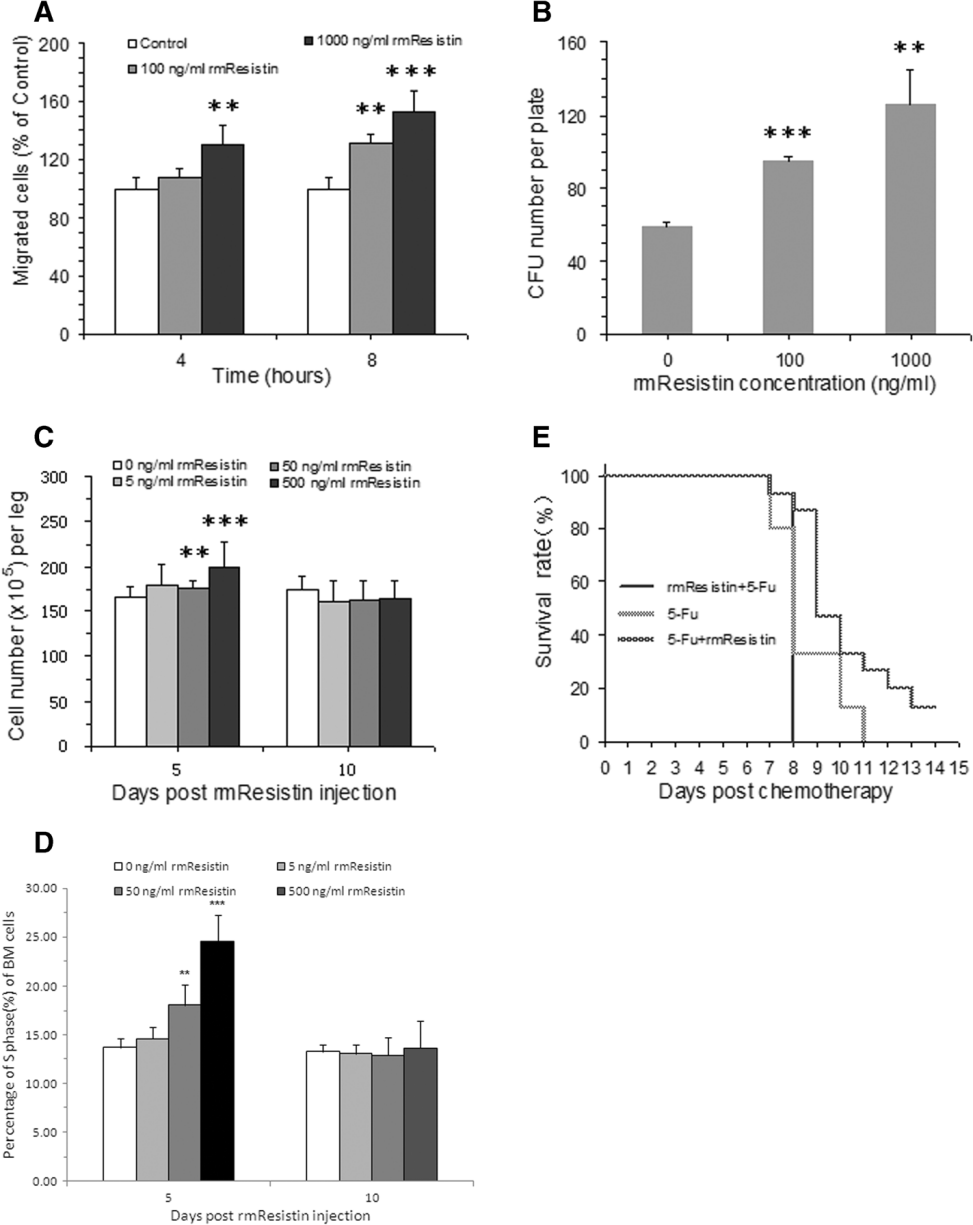
Results of bioactivity determination and hemotopoiesis investigation of rmRetn on mouse BM. **a**: Migration of MAECs was accelerated by rmRetn at 100 or 1000 ng/ml after incubation of 8 h compared with control (*p* = 0.0049 and *p* < 0.001, *n* = 3); After incubation of 4 h, 100 ng/ml group didn’t exhibit significant difference from control group (*p* > 0.05, *n* = 3), while migrated number of 1000 ng/ml group was extremely significant compared with control group (*p* = 0.0034, *n* = 3). **b**: CFU numbers of BM per plate in the presence of rmRetn (100 and 1 000 ng/ml) were significantly increased compared with control (*p* < 0.001 and *p* = 0.0087, *n* = 3). **c**: Total BM cell numbers of normal mice were increased significantly after continuous administeration of rmRetn for 5 days, twice per day, at the dose of 50 and 500 μg/kg compared with control (PBS) group (*p* < 0.001 and *p* = 0.0036 respectively, *n* = 16) except for 5 ng/kg group (*p* > 0.05, *n* = 16). While the difference didn’t exist at day 10 after continuous administration of rmRetn at all the three doses of 7 days (*p* > 0.05, *n* = 16). **d**: Percentage of S phase of BM cells was raised largely after continuous administeration of rmRetn for 5 days, twice per day, at the dose of 50 and 500 μg/kg compared with control (PBS) group (*p* < 0.001 and *p* = 0.0036 respectively, *n* = 16) except for 5 ng/kg group (*p* > 0.05, *n* = 16). While the difference didn’t exist at day 10 after continuous administration of rmRetn at all the three doses of 7 days (*p* > 0.05, *n* = 16). **e**: Pretreatment and postreatment of rmRetn displayed thoroughly reverse effects on mice injected with 5-fluorouracil (300 mg/kg). Adminsteration of rmRetn post 5-fluorouracil treatment prolonged the life time of animals and saved two mice from the chemotoxicity of 5-Fluorouracil compared to the zone survival in control group (with only 5-fluorouracin injected) (*p* = 0.024, *n* = 15); However, the animals with mRetn pretreatment had a shorter life span than the control animals. Although the difference between pretreatment group and control group wasn’t statistically meaningful (*p* > 0.05, *n* = 15), the difference between pre- and post-treatment group was statistically meaningful (*p* < 0.001, *n* = 15); The result indicated that post-treatment of rmRetn could protect mice from the toxicity of 5-fluorouracil and increased their survival rate

Both human and mouse Retn have been reported to be able to promote endothelial cell activation [21, 22], proliferation and migration [16, 23]. Here, a transwell experiment was designed to investigate the migration promoting bioactivity of rmRetn on MAECs, and the concentrations of rmRetn used were referred to the previous report [16]. The cell migration results were shown in Fig. 4a. Compared to the control, rmRetn at both 100 and 1000 ng/ml attracted more cells to transfer into the bottom chamber. The groups showed significant differences between each other after 8 h. As a whole, rmRetn promoted MAECs to migrate in both time- and dose-dependent manners and the effects were exact and specific. The results indicated obviously that the prepared rmRetn possessed the bioactivity of mRetn and was suitable for animal experiments.

### rmResisitin increased bone marrow CFU of mice in vitro

When cultured in medium with rmRetn, mouse bone marrow cells responded significantly to the protein at concentrations of 0.1 μg/ml (*p* < 0.001, *n* = 3) and 1 μg/ml (*p* < 0.01, *n* = 3), compared to the control group with PBS (Fig. 4b). Moreover, the effect was dose-dependent. The results indicated undoubtedly that rmRetn could activate the bone marrow cells and promote their proliferation, which means that the bone marrow cells were motivated and entered into active cell cycles from the quiescent state. Thus, if subjected to rmRetn at a suitable concentration, mouse BM cells might increase to a higher level than normal, and the deduction was proved to be true in the following experiment.

### rmRetn increased hematocytes of normal mice in vivo

As expected, the number of total bone marrow cells was significantly increased on the fifth day after the rmRetn injections at doses of 50 and 500 ug/kg/day (*p* < 0.05, *n* = 16; *p* < 0.01, *n* = 16; Fig. 4c). Although the cell number at the lowest dose (5 μg/kg/day) did not differ meaningfully from the control group, the increasing averages of cell number demonstrated that the hemo-stimulating effect was probably in a dose-dependent manner. Additionally, the percentage of S phase of bone marrow cells was raised distinctly after the rmRetn injections (*p* < 0.05, *n* = 16; *p* < 0.01, *n* = 16; Fig. 4d). On day 10 post rmRetn injection, the BM cell number and percentage of S phase did not show any change and this was might due to the elimination of rmRetn in the blood circulation and the BM cells went back to being quiescent again.

### rmRetn increased mouse survival from acute lethal toxicity of 5-Fluorouracil

Since the hematopoiesis of BM was motivated by rmRetn, it could probably protect mice from the hematoxity induced by chemotherapy agents, just as G-CSF could protect mice neutrophils from chemotherapy. When treated at the lethal dose (300 mg/kg) as previously used in our labarotory [20], all mice died on day 11, namely 5 days post chemotherapy. However, admisnistration of rmRetn following the 5-flurouracil injection, reduced the mortality rate of mice, and prolonged the survival period of the animals. The survival rates of these two groups differed significantly (*p* < 0.05, *n* = 15; Fig. 4e). Meanwhile, a group of mice were especially useful here to verify the hemo-stimulating effect from the other side. Pretreatment of rmRetn might motivate BM cells before chemotherapy, and these active cells would be more sensitive and feasible to chemotherapic agents, exacerbating the toxicity and inducing more lethality. And the results confirmed the hypothesis as shown in Fig. 4e. Although the difference of the survival rate between the pretreatment group and the control chemotherapy group was not significant (*p* > 0.05, *n* = 15), the survival period of animals in the chemotherapy group was obviously prolonged. When the rmRetn pretreatment group was compared with the rmRetn posttreatment group, the survival rate was extremely different (*p* < 0.001, *n* = 15). These results indicated that rmRetn might be a protective agent against the acute lethal toxicity of chemotherapy for mice.

## Discussion

In this study, we attempted to verify the hypothesis derived from the genechip hybridization and to determine whether mouse Retn could exert effects on hematopiesis in mice. Firstly, a cost-effective and feasible producing process was designed to prepare enough protein for the animal experiments. Expression, purification, denaturation, and renaturation of rmRetn were all utilized in the process successively to prepare the soluble protein. Ion exchange chromatographies were applied to remove impurities. The advantages of recovering a biologically active protein from inclusion bodies, such as highly enriched targeted protein, is the resistance to proteolysis by E. coli proteases and the convenience to isolate from host strain. More importantly, it facilitates highly pure protein production [24, 25]. Thus, a successful strategy was employed here to recover enough pure rmRetn with low level of endotoxin for use.

As to animal studies, 5-FU model is one of the most common model for researching BM suppression and regeneration post chemotherapy. In our work, it suits well. The possible role of Retn in hematopoiesis is suggested by our results: (1) the temporal induction of Retn expression in BM cells inversely suits the BM cellularity after 5-Fu induced suppression and regeneration. This suggests a role of Retn involving BM homeostasis; (2) elevation of Retn by administration of recombinant protein significantly increases the numbers of HPCs (hematopoietic progenitor cells) tested by cell proliferation-stimulating activity (CFU) test in vitro, and circulating Retn by injection of recombinant protein significantly raises the numbers of BM cells in vivo through promoting the progression of BM cell cycle; and (3) as a consequence of its promotion of cell cycle entry into S phase, elevation of Retn plasma levels after 5-Fu treatment magnificently alleviates mouse mortality.

In summary, the hypothetical hematopoiesis of mRetn was confirmed by BM CFU assay, BM cell proliferation assay and survival assay in vitro and in vivo [26]. All the results of these assays demonstrated that rmRetn might help accelerate BM recovery from 5-fluorouracil through activating BM cells, which suggested that Retn might play a protective role during chemotherpy in clinic. Compared to a supressive agent of BM hematopoiesis used prior to chemotherapy [20] such as G-CSF [27], mRetn takes a direct effect on BM cells post chemotherapy, which might take a more significant effect compared to the former. As one of the most serious side effects of cancer chemotherapy and radiotherapy, hematopoietic system impairs often leads to neutropenia which is currently treated with G-CSF [28] as the first resort. Therefore, the hematopoiesis of mRetn will be our next emphasis. Of couse, the mechanism of motiviating BM cells is still mysterious and will be focused on in the following work.

The direct evidence was rarely observed about hematopoiesis of Retn, while indrect evidences, including our genechip data, have been reported repeatedly. Retn plays roles mainly in inflammation, glucose metabolism and insulin resistance [29, 30]. Recently, Retn was proved to be able to induce human endothelial cell proliferation, migration and in vitro angiogenesis through activating ERK1/2 and p38 [23]. These findings provide a direct link between Retn and angiogenesis. Furthermore, both Retnlg, an important member of the Retn family, and human RETN are expressed in myeloid cells with the highest levels found in the bone marrow [11, 31]. The previous information delivers a message that Retn joins not only in the vascular lesion formation, but also in haematopoietic cell development. Until the mechanisms of the stimulating effects on the BM cells of rmRetn are identified, the utilization of Retn for the regulation of whole blood circulation system, and for cancer chemotherapy and radiotherapy can not be understood.

## Conclusions

In summary, we prepared a sufficient amount of rmRetn with high-purity, high biological activities and low endotoxin level by a feasible and economic way, which totally matches the native mouse Resistin in sequence with no additional tag, and presented new effects of rmResistin then verified our hypothesis that mRetn can activate BM cells to resist the toxicity of chemopeutical agents, which was derived from our genechip data. It was the first time that the possible hematopoiesis effect of mRetn was identified and proven, which may reduce the side effects induced by chemotherapy and help cancer patients survive from chemotherapy and improve their quality of life. As to why rmRetn can help accelerate the HSC division of mice still needs to be investigated, which we will carry out in the further.

Most importantly, we hope that, with our method, the ability to produce milligram quantities of bioactive protein from E. Coli and the investigation of protein function will facilitate the study of biochemical and pathological roles of Resistin.

## Abbreviations

5-Fu: 5-fluorouracil
BM: bone marrow
CFU: colony-forming units
HPCs: hematopoietic progenitor cells
HPLC: high performance liquid chromatography
PB: peripheral blood
Retn: Resistin
rmRetn: recombinant mouse Resistin
SDS-PAGE: sodium dodecyl sulfate polyacrylamide gelelectrophoresis
